# A novel preclinical model of craniospinal irradiation in pediatric diffuse midline glioma demonstrates decreased metastatic disease

**DOI:** 10.3389/fonc.2023.1105395

**Published:** 2023-04-12

**Authors:** Aaron J. Knox, Benjamin Van Court, Ayman Oweida, Elinor Barsh, John DeSisto, Patrick Flannery, Rakeb Lemma, Hannah Chatwin, Rajeev Vibhakar, Kathleen Dorris, Natalie J. Serkova, Sana D. Karam, Ahmed Gilani, Adam L. Green

**Affiliations:** ^1^ Morgan Adams Foundation Pediatric Brain Tumor Research Program, Department of Pediatrics, University of Colorado School of Medicine, Aurora, CO, United States; ^2^ Department of Radiation Oncology, University of Colorado School of Medicine, Aurora, CO, United States; ^3^ Children’s Hospital Colorado, Aurora, CO, United States; ^4^ Department of Radiology, University of Colorado School of Medicine, Aurora, CO, United States; ^5^ Department of Pathology, University of Colorado School of Medicine, Aurora, CO, United States

**Keywords:** diffuse midline glioma, craniospinal irradiation, patient-derived xenograft, metastatic disease, pediatric

## Abstract

**Background:**

Diffuse midline glioma (DMG) is an aggressive pediatric central nervous system tumor with strong metastatic potential. As localized treatment of the primary tumor improves, metastatic disease is becoming a more important factor in treatment. We hypothesized that we could model craniospinal irradiation (CSI) through a DMG patient-derived xenograft (PDX) model and that CSI would limit metastatic tumor.

**Methods:**

We used a BT245 murine orthotopic DMG PDX model for this work. We developed a protocol and specialized platform to deliver craniospinal irradiation (CSI) (4 Gy x2 days) with a pontine boost (4 Gy x2 days) and compared metastatic disease by pathology, bioluminescence, and MRI to mice treated with focal radiation only (4 Gy x4 days) or no radiation.

**Results:**

Mice receiving CSI plus boost showed minimal spinal and brain leptomeningeal metastatic disease by bioluminescence, MRI, and pathology compared to mice receiving radiation to the pons only or no radiation.

**Conclusion:**

In a DMG PDX model, CSI+boost minimizes tumor dissemination compared to focal radiation. By expanding effective DMG treatment to the entire neuraxis, CSI has potential as a key component to combination, multimodality treatment for DMG designed to achieve long-term survival once novel therapies definitively demonstrate improved local control.

## Introduction

Diffuse midline gliomas (DMG) are a highly aggressive subtype of pediatric high-grade glioma. These tumors are universally fatal, with a median overall survival of less than 1 year. They account for 10-20% of pediatric central nervous system (CNS) malignancies and are the leading cause of pediatric CNS tumor-related death (1).

Focal radiation therapy (RT) remains the only therapeutic approach that has been shown definitively to lengthen DMG survival, although it usually only delays progression of the tumor by a few months. There has been minimal improvement in the prognosis of DMG due to the failure of previous clinical trials (2). Focal RT has been the standard RT field because focal progression in the pons or other primary site is usually responsible for death. However, DMG demonstrates high rates of leptomeningeal dissemination at diagnosis, during treatment, and at autopsy (3, 4). With recent or current early trials of techniques allowing localized, primary tumor-directed delivery of chemotherapy agents, including convection-enhanced delivery (CED) (5) and focused ultrasound with microbubbles (6), the potential for prolonged local control of DMG is becoming more realistic; however, some patients are now experiencing metastatic progression while their primary tumor is responding to these treatments. Thus, controlling metastatic DMG has become a pressing challenge in the fight to achieve long-term survival in this disease. Craniospinal irradiation (CSI) is a sensible modality to consider, since DMG is sensitive to RT, and CSI is crucial to survival in multiple other pediatric brain tumors with metastatic potential, such as medulloblastoma and atypical teratoid/rhabdoid tumor.

In the present study, we aimed to investigate CSI as a therapeutic approach to control metastatic disease across the neuraxis in an orthotopic patient-derived xenograft (PDX) model of DMG. We hypothesized that we could feasibly study DMG preclinically using a PDX model, and that CSI would limit metastatic disease in this model compared to focal RT, although it would not extend survival alone.

## Methods

### PDX model and radiation treatment

Patient-derived mouse models of DMG were generated using immunocompromised Foxn1^nu^/Foxn1^nu^ mice. 200,000 luciferase-expressing BT245 DMG cells were injected intracranially into the right pons of 6–8 week-old mice as previously described (7). Tumor-bearing mice were randomized to one of three treatment groups: no radiation, pontine-directed focal radiation, or CSI with a focal pontine boost. A relatively large group size of 12 was chosen based on known incomplete penetrance of metastatic tumor development in the model. 23 days after tumor cell injection, mice began the assigned radiation treatment. The focal group received 4 Gy radiation to the pons on four consecutive days, while the CSI group received two days of 4 Gy CSI and two days of 4 Gy pons-directed radiation. These regimens were chosen in discussion with the radiation oncology team, led by Dr. Karam, to be feasible in mice and representative of the fairly even CSI and boost RT doses used in pediatric patients. Radiotherapy was performed using the X-RAD SmART image-guided irradiator (Precision X-Ray Inc., Branford CT) at 225kVp, 20mA with 0.3 mm Cu filter. Mice were anesthetized with isoflurane and positioned supine in a custom 3D-printed holder. Craniospinal irradiation was delivered in two pairs of opposing lateral beams, using a custom Cerrobend collimator. Radiation dosimetry was performed using a combination of Monte-Carlo simulations from SmART-ATP treatment planning software (SmART Scientific Solutions, Maastricht, The Netherlands) and Gafchromic EBT3 dosimetric film (Ashland Global, Wilmington, DE). Mice were monitored for onset of weight loss, mobility, and neurological symptoms and were sacrificed when these became apparent. At necropsy, tumors, brains, and spines were formalin fixed and hematoxylin/eosin (H&E) stained. The experiment was repeated for validation.

### Animal imaging (Bioluminescence and MRI)

Tumor growth was monitored weekly by bioluminescence imaging (BLI) on luciferase-expressing cells using IVIS Spectrum (PerkinElmer, Waltham, MA). At the end of the study, the animals with the BLI-confirmed tumors underwent a multiparametric MRI session consisting of a high-resolution, T2-weighted turboRARE 3D-MRI (for tumor localization and volume, in-plane resolution 52 microns), a FLAIR MRI (for inflammation and edema), and diffusion-weighted imaging (DWI, for tumor cellularity and parenchymal edema) protocols (8). All MRI was performed on a Bruker 9.4 Tesla BioSpec MRI scanner (Bruker Medical, Billerica, MA) using a mouse head phase-array coil. All MR acquisition and image analysis was performed using Bruker ParaVision NEO v.3.1. software.

### Analysis of tumors and metastases

Mouse survival was measured by the Kaplan-Meier method and compared by log-rank test. The sizes of primary tumors, regional metastasis, and spinal metastasis were analyzed by a pediatric neuropathologist blinded to treatment group. Primary tumors and each area of metastases (brain leptomeninges, lateral ventricles, and spinal cord) were graded on a 0 (no evidence of tumor) to 3 (severe metastatic involvement) scale.

## Results

### DMG model and RT fields

**Figure 1:**
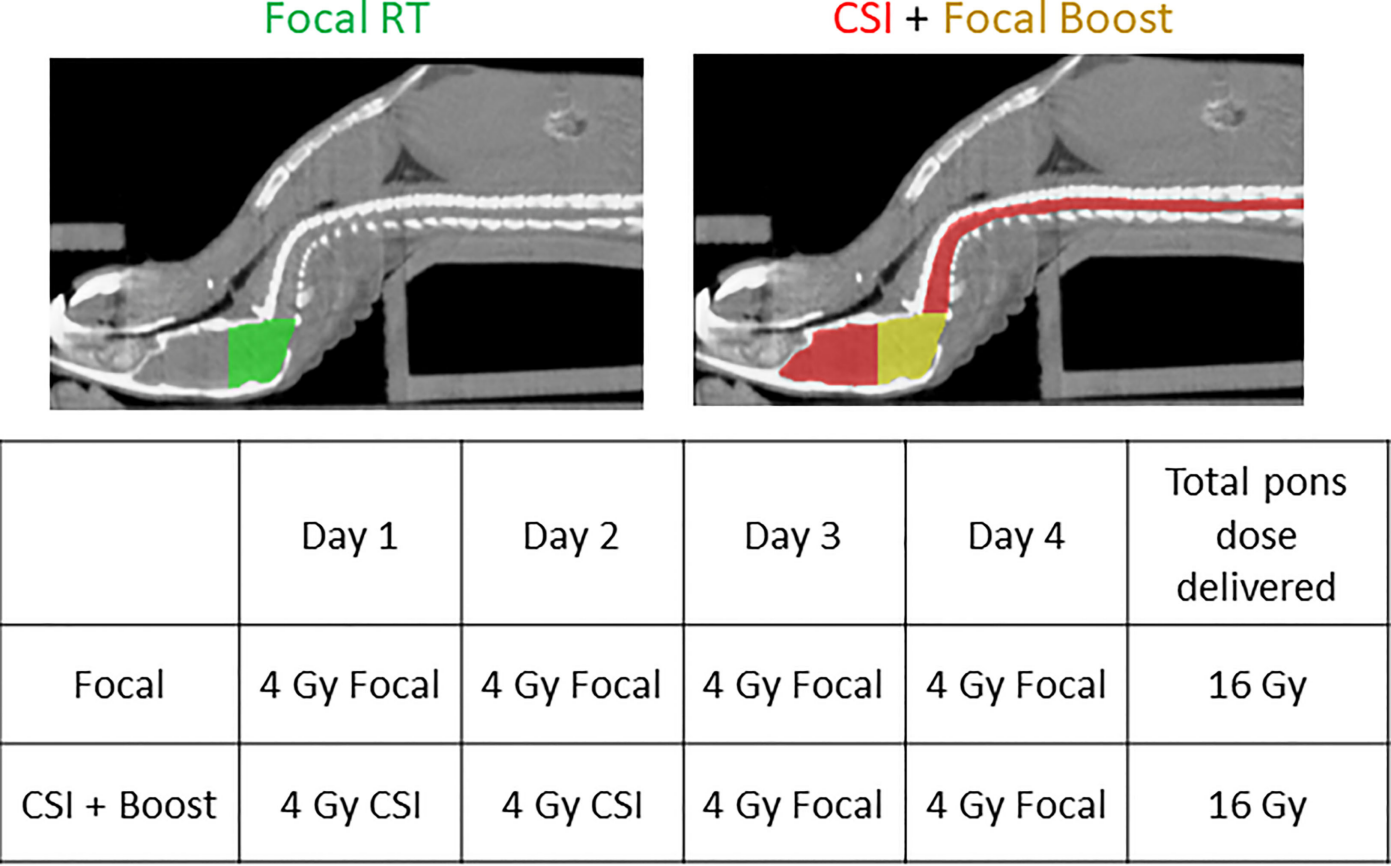
Planning CTs demonstrating positioning of the mouse on the custom platform and RT fields for the two RT groups, along with the RT schedule for both groups.

For our assessment of radiation fields in controlling DMG metastasis, we chose the BT-245 DMG model, which originated from a pediatric thalamic DMG collected at initial resection (7, 9). We built a custom 3D-printed platform to facilitate murine focal pontine and CSI+boost radiation delivery while avoiding RT to other thoracic and abdominal organs (**Figure 1**).

### Treatment, tumor monitoring, and survival

**Figure 2:**
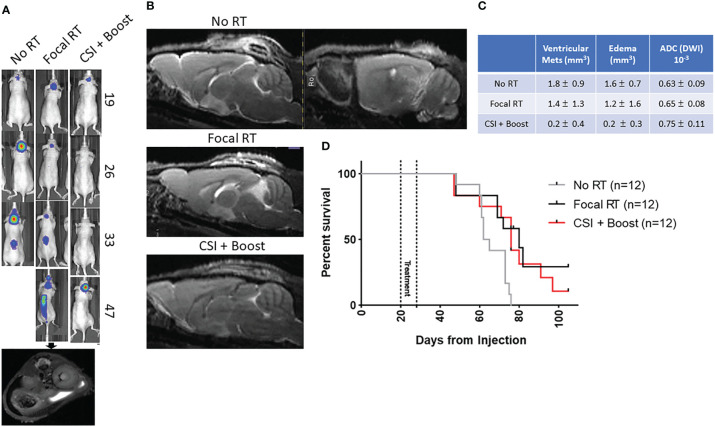
**(A)** Representative BLI for each group with day from tumor injection on the right, with a representative MRI confirming spinal tumor spread; **(B)** Representative sagittal T2 MRI images for the no RT (with T1 as well), focal RT, and CSI+boost groups show abnormal leptomeningeal and ventricular signal in the no RT/focal RT mice brains with no apparent metastases in the CSI+boost mouse brain; **(C)** Quantitative MRI endpoints for ventricular metastatic lesion volumes (T2-MRI), edema volumes (FLAIR, DWI), and apparent diffusion coefficients (DWI) showing mean values +/- standard deviation; **(D)** Kaplan-Meier curve for the three RT groups show prolonged survival for focal RT and CSI+boost groups compared to no RT (p=0.01 for both) but no difference in survival between the groups receiving RT (p=0.48).

23 days after tumor cell injection, when the first mice had developed signs of tumor by BLI, mice were randomized to one of three RT conditions and began treatment: no RT, focal pontine RT 4 Gy x4 days, and CSI 4 Gy x2 days + boost 4 Gy x2 days. Details of the radiation dose mapping and delivery are shown in **Supplemental Figure 1**. 12 mice were randomized to each treatment group. Mice were monitored weekly by whole-body BLI (**Figure 2A**) and by brain and spine MRI at a single time point approximately one week after the end of the treatment period (**Figure 2B**). Representative BLI images show steady primary tumor growth in the no RT mouse followed by spinal metastatic progression and death. In the focal RT group mouse, the primary tumor responds initially to RT, but metastatic disease develops, with growth of both primary tumor and spinal metastases before death. In the CSI+boost mouse, the primary tumor improves with radiation but eventually recurs, without the development of metastases. Representative MRI images show abnormal signal consistent with brain leptomeningeal and ventricular metastatic disease in the focal RT mouse but not in the CSI+boost mouse. Quantitative MRI endpoints for DMG treatment response are presented in **Figure 2C**. Metastatic invasion into the ventricles was partially prevented in the CSI+boost animals (p<0.0001 compared to no RT and p=0.006 compared to focal RT by t-test). In both the focal RT and CSI+boost groups, intracranial lesions revealed apparent diffusion coefficients (ADC from DWI) that were slightly higher as compared to the no-RT group, indicating lower cellularity/proliferation index after RT. Mice receiving focal RT and CSI+boost both showed prolonged survival in comparison to no RT mice (p=0.01 for each group vs. no RT), but there was no survival difference based on RT field (**Figure 2D**). This finding indicates that mice were likely dying of local tumor progression and not metastatic disease.

### Histopathological assessment

**Figure 3:**
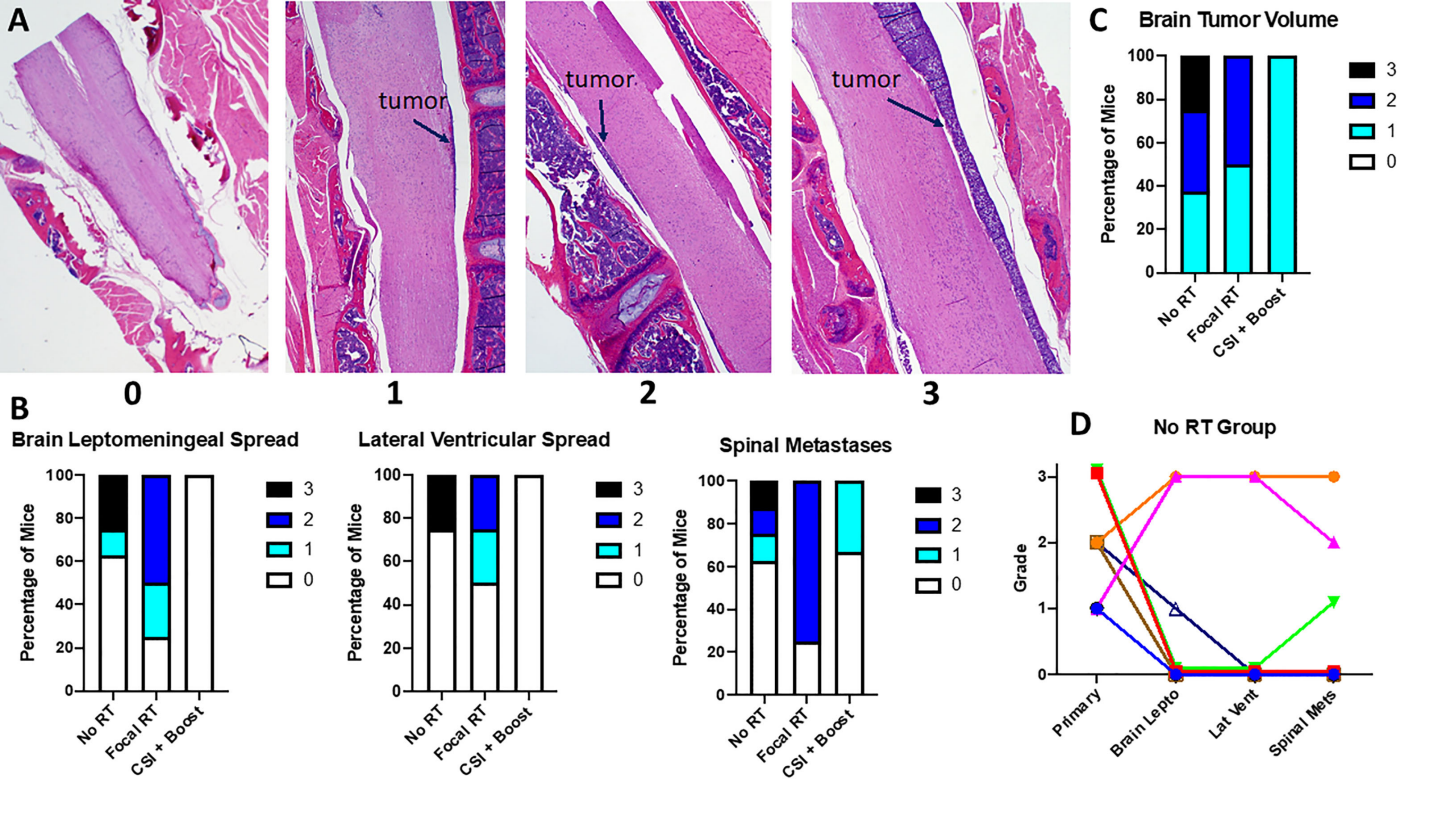
**(A)** Sagittal spinal cord images show examples of each gradation of metastatic disease; **(B)** Grading of metastases across locations for all mice by treatment group; **(C)** Grading of primary tumor volume for all mice by treatment group; **(D)** Correlation between primary tumor volume and metastatic gradation at each site measured for the no RT group; each color/shape represents a different individual mouse.

Mouse brains and spines were collected at necropsy and assessed for tumor involvement by a pediatric neuropathologist (Dr. Gilani) blinded to treatment group. Areas of metastatic tumor involvement on H&E staining are shown in **Supplemental Figures 2A, B**, along with an MRI image showing correlation of MRI and histopathological findings. Dr. Gilani graded primary (pontine) tumor volume and metastatic tumor involvement on a 0 (no tumor) to 3 (severe tumor involvement) scale (**Figure 3A**). While not all mice developed metastatic tumor, mice from the no RT and focal RT groups were more likely to have metastatic involvement overall and more severe metastases across all areas assessed (**Figure 3B**). While there were also differences in primary tumor volume between treatment groups (**Figure 3C**), primary tumor grade did not appear to correlate with metastatic tumor grade in the no RT group (**Figure 3D**).

## Discussion

Using a murine orthotopic xenograft model of DMG, we identified CSI as a therapeutic modality capable of limiting metastatic disease throughout the CNS compared to focal RT, the current standard-of-care therapy in DMG. This brief report represents the first preclinical assessment of CSI in DMG and raises this treatment modality as a potential contributor to a combination approach in this disease. This approach is necessitated by a developing “good” problem in DMG, that more prolonged local tumor control is potentially becoming more feasible with the introduction of experimental treatment modalities allowing focal delivery of chemotherapy, leading to the emergence of clinically apparent disease dissemination in some patients as their survival has been prolonged.

In our study, mice in the focal RT and no RT groups showed similar levels of metastatic disease. Since the only difference between these groups was pontine RT, this suggests that metastatic tumor was seeded throughout the CNS early in the disease process instead of continuing to disseminate from the primary tumor, as in this case we would expect decreased metastatic disease with focal RT. BLI tended to show spinal metastases only later, however, which matches what has been seen in patients: metastatic DMG is less frequent by MRI at diagnosis than later in treatment (3) but can often be detected at diagnosis by more sensitive assays like circulating tumor DNA, the use of which is experimental but emerging in this disease (4). The timing of treatment in this model, at first detection of abnormal signal by imaging, is necessary in mice given the need for sacrifice at symptom development, but it presents a potential issue in the translation of our findings to patients, who do not present until developing symptoms. However, the findings from our work and human studies (4), both showing metastatic tumor present from first detection of the primary tumor, suggest that CSI delivered at the time of diagnosis would be effective.

Weaknesses of the current study include the use of only one radiation dosing schedule and one model, which harbors both H3K27M and *TP53* somatic mutations; *TP53* mutation is actually associated with radiation resistance in DMG (10), so we might expect an even more profound impact of radiation in *TP53-*wild type tumors, and future work on CSI should explore a variety of DMG models. Another weakness is the inability to be sure that the pattern of metastatic disease development in this model matches that seen in patients. However, the injection of cells directly into the pons (as opposed to the ventricle), the variability of metastatic disease development, and the findings on histopathology all suggest accurate modeling of true metastases akin to those seen in patients. Strengths of the study include the multiple assessments of metastatic disease by dual imaging modalities and histopathology, the grading of metastases by a neuropathologist blinded to treatment group, and the novel approach of delivering CSI to mice through a custom-made platform to minimize toxicity.

As expected, CSI did not produce a survival benefit over focal RT in our study, as mice still died of their primary tumor with radiation alone. This again matches what has been seen in patients: RT alone is a palliative measure only in DMG that can only delay tumor progression, including in the few documented patients in which CSI has been given for metastatic tumor (11, 12), especially since even maximal CSI doses are lower than doses that can be delivered to the primary tumor. However, these studies did indicate temporary efficacy against these metastases, and combined with our findings, suggest that CSI has potential as a component of combination, multimodality therapy against DMG to control the tumor dissemination likely to become clinically apparent as treatment measures directed at the focal tumor become more effective at prolonged local control. Our methods and findings provide the groundwork for CSI to be incorporated into future preclinical studies and clinical trials. Combination approaches (13) addressing both the primary tumor through focal RT and targeted delivery of chemotherapy and/or immunotherapy (14, 15), as well as disseminated tumor through CSI and systemic chemotherapy and/or immunotherapy, have the potential to produce long-term control of DMG.

## Data availability statement

The original contributions presented in the study are included in the article/**Supplementary Material**. Further inquiries can be directed to the corresponding author.

## Ethics statement

The animal study was reviewed and approved by Institutional Animal Care and Use Committee, University of Colorado Anschutz Medical Campus.

## Author contributions 

ALG conceptualized and planned the project, with contributions from RV and KD. ALG, AK, BC, AO, and SK created the radiation plan. AK, BVC, and AO carried out the experiments. NJS led animal imaging (BLI and MRI). AG led histopathological analysis. AK, AG, and ALG led data analysis, assisted by JD, PF, RL, and HC. EB, AK, and ALG wrote the manuscript. All authors contributed to the article and approved the submitted version.

## Supplementary material

Supplementary Figure 1
**(A)** Screenshot from SmART-ATP treatment planning software showing dose distribution for focal irradiation. Approximate tumor location is shown in green; **(B)** Dose-volume histogram for spine, brain, and pons, based on simulated focal irradiation; **(C)** Illustration of the two pairs of opposing lateral beams used for CSI on the fluoroscopy image on an x-ray image of a mouse on the custom platform; **(D)** Simulated dose distribution for opposing lateral beams through the spine, showing roughly uniform dose for all soft tissue in the field. Note that the distribution simulated for spinal irradiation is not for the exact collimator used to treat these mice; a dose-rate correction factor for the custom collimator based on Gafchromic film dosimetry was used to determine the appropriate beam times from the simulation.

Supplementary Figure 2
**(A)** Example images showing metastatic deposits in various areas of the brain on histopathology, with an axial MRI showing correlating leptomeningeal metastases from the same mouse; **(B)** Example images showing metastatic tumor at different levels of the spine on sagittal sections
