# Supplementary figures and images for: A novel preclinical model of craniospinal irradiation in pediatric diffuse midline glioma demonstrates decreased metastatic disease
Source: Front Oncol. 2023 Apr 12;13:1105395. doi: 10.3389/fonc.2023.1105395 (PMC10132465; doi:10.3389/fonc.2023.1105395)

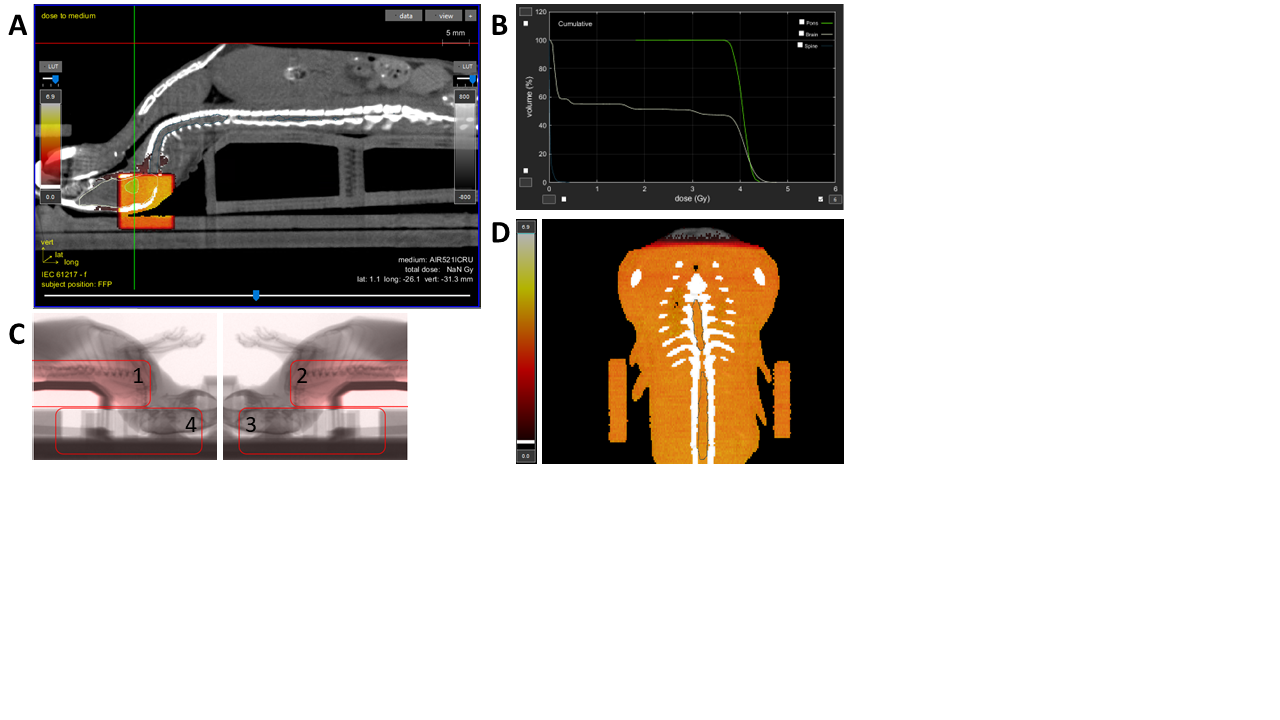

Supplement: Supplementary Figure 1 — (A) Screenshot from SmART-ATP treatment planning software showing dose distribution for focal irradiation. Approximate tumor location is shown in green; (B) Dose-volume histogram for spine, brain, and pons, based on simulated focal irradiation; (C) Illustration of the two pairs of opposing lateral beams used for CSI on the fluoroscopy image on an x-ray image of a mouse on the custom platform; (D) Simulated dose distribution for opposing lateral beams through the spine, showing roughly uniform dose for all soft tissue in the field. Note that the distribution simulated for spinal irradiation is not for the exact collimator used to treat these mice; a dose-rate correction factor for the custom collimator based on Gafchromic film dosimetry was used to determine the appropriate beam times from the simulation. [file Image_1.tif]

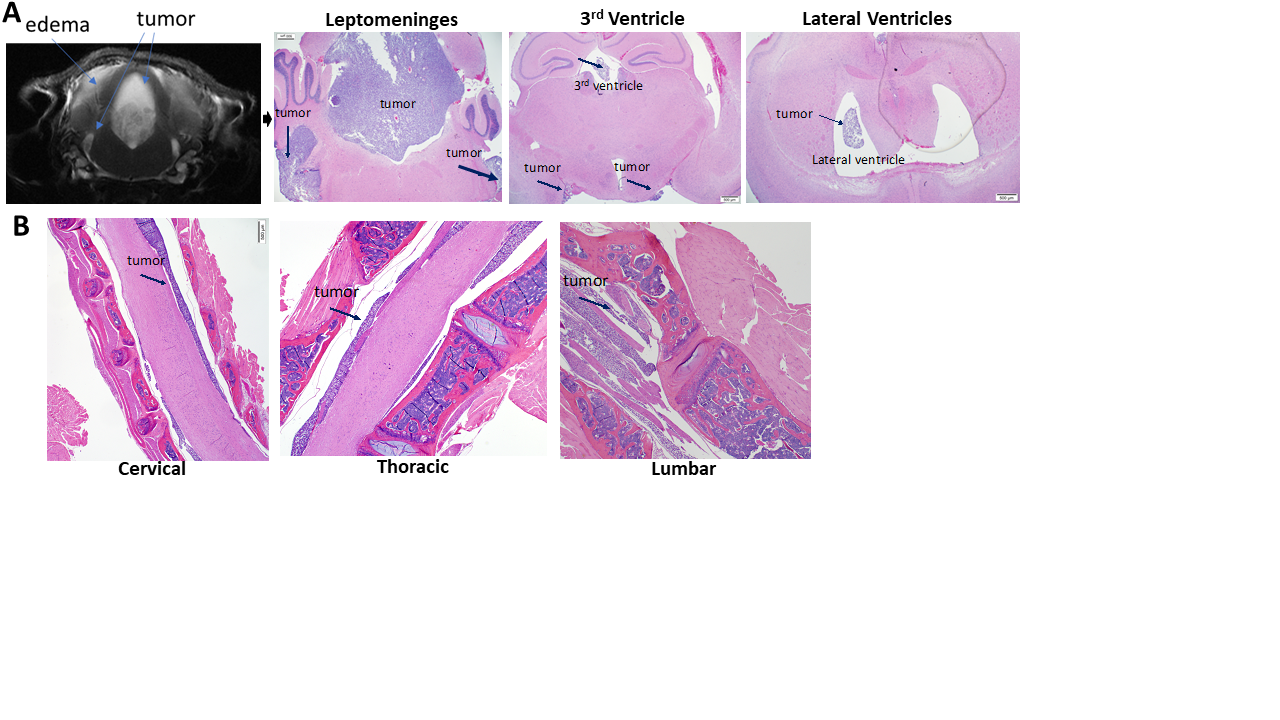

Supplement: Supplementary Figure 2 — (A) Example images showing metastatic deposits in various areas of the brain on histopathology, with an axial MRI showing correlating leptomeningeal metastases from the same mouse; (B) Example images showing metastatic tumor at different levels of the spine on sagittal sections [file Image_2.tif]
